# Translocation Dynamics of High-Internal Phase Double Emulsions in Narrow Channels

**DOI:** 10.1021/acs.langmuir.1c01026

**Published:** 2021-07-22

**Authors:** Andrea Montessori, Adriano Tiribocchi, Michał Bogdan, Fabio Bonaccorso, Marco Lauricella, Jan Guzowski, Sauro Succi

**Affiliations:** † Istituto per le Applicazioni del Calcolo CNR, Via dei Taurini 19, Rome 00185, Italy; ‡ Center for Life Nanoscience at la Sapienza, Istituto Italiano di Tecnologia, Viale Regina Elena 295, Rome 00161, Italy; § Institute of Physical Chemistry, Polish Academy of Sciences, Kasprzaka 44/52, Warsaw 01-224, Poland; ∥ Dipartimento di Fisica, Università degli Studi di Roma “Tor Vergata”, Via della Ricerca Scientifica 1, Rome 00133, Italy; ⊥ Institute for Applied Computational Science, Harvard John A. Paulson School of Engineering and Applied Sciences, Cambridge, Massachusetts 02138, United States

## Abstract

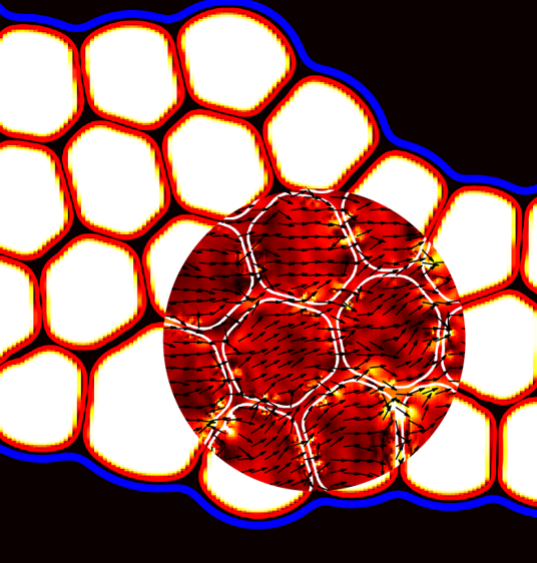

We numerically study the translocation dynamics of double emulsion drops with multiple close-packed inner droplets within constrictions. Such liquid architectures, which we refer to as HIPdEs (high-internal phase double emulsions), consist of a ternary fluid, in which monodisperse droplets are encapsulated within a larger drop in turn immersed in a bulk fluid. Extensive two-dimensional lattice Boltzmann simulations show that if the area fraction of the internal drops is close to the packing fraction limit of hard spheres and the height of the channel is much smaller than the typical size of the emulsion, the crossing yields permanent shape deformations persistent over long periods of time. Morphological changes and rheological response are quantitatively assessed in terms of the structure of the velocity field, circularity of the emulsion, and rate of energy dissipated by viscous forces. Our results may be used to improve the design of soft mesoscale porous materials, which employ HIPdEs as templates for tissue engineering applications.

## Introduction

**Figure 1:**
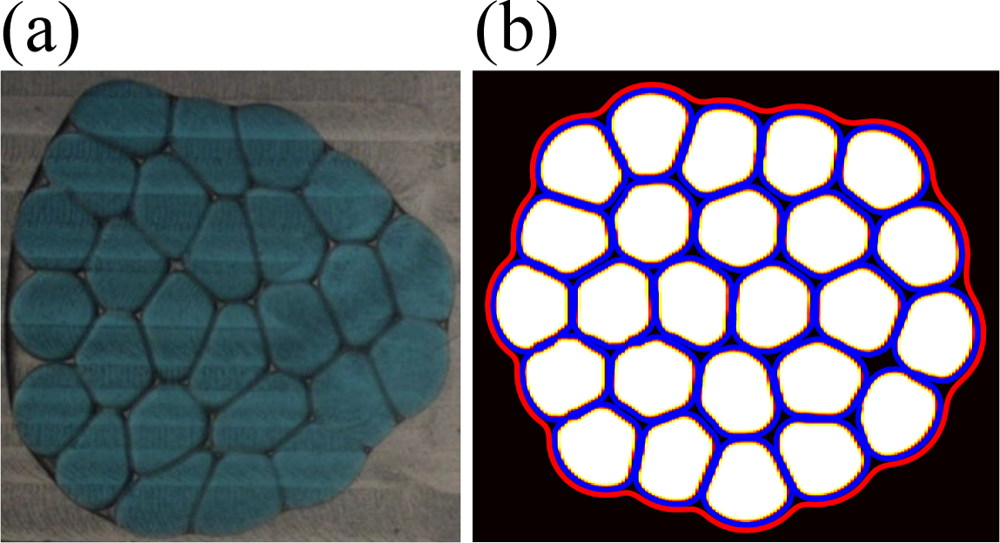
(a) Experimental result of a HIPdE made of approximately monodisperse drops encapsulated within a larger droplet. Three Newtonian liquids are used to formulate the mixture. The continuous fluid is made from a 1% solution of PFPE–PEG–PFPE in fluorinated oil FC40, and the lubricating fluid of the emulsion is a solution of 5cSt silicon oil, hexadecane, and SPAN80 in proportions 70:30:1. Finally, the innermost phase is water-painted with euroglaucine. Estimated values of viscosity of continuous and lubricating fluid are approximately 4.0 mPa·s, while the value of the innermost phase is 1.0 mPa·s. (b) Example of a HIPdE obtained by LB simulations. It is made of a dispersed droplet phase (white), an inter-droplet lubricating phase (blue) surrounded by a fluid interface (red), and an external fluid (black). The compact foam-like arrangement results from the translocation of the emulsion within a thin channel, followed by a slow relaxation in the downstream reservoir. See the section Results for more details.

High-internal phase emulsions (HIPEs) are two-phase systems, in which a very high volume fraction of dispersed droplets, typically well above the close packing limit for spheres, arrange in a tightly packed, strongly deformed configuration.^1^ An aliquot of such materials suspended in an external fluid forms a double-emulsion structure with highly packed “cores” encapsulated in a single “shell”, a structure that we further refer to as high-internal phase double emulsion (HIPdE).^2−14^ Like in HIPEs, the cores generally occupy an area fraction ϕ larger than the close packing limit for hard spheres (ϕ ≃ 0.74); thus, they acquire a polyhedral, rather than perfectly spherical, shape (see Figure 1). A typical example of such a material is given by monodisperse aqueous droplets packed within an encapsulating shell containing an immiscible oil phase, in turn immersed within a bulk aqueous phase. This mixture is generally stabilized using a suitable surfactant adsorbed onto the water–oil interfaces.^1,12,15−18^

The resulting compartmentalized structure is highly desirable in numerous applications, ranging from cosmetic products^9,19^ to functional foods,^20−22^ and can be also used as a template for the fabrication of porous scaffolds for tissue engineering.^1,11,23−25^ In addition, such soft granular aggregates provide an ideal tool to mimic the behavior of multicellular spheroids in confined environments or under external stresses,^58^ such as clusters of tumor cells circulating within capillary-sized vessels^26−28^ or fusing cell aggregates employed to fabricate large living constructs in tissue engineering applications.^29^

Densely packed emulsions exhibit intriguing and complex mechanical properties, in stark contrast with the characteristics of simple viscous liquids they are made of (water and oil). In particular, depending on the magnitude and rate (timescale) of externally applied stresses, the emulsions may behave either as a fluid (high stresses, long timescales) or as a solid (small stresses, short timescales).^30^ These features are of particular relevance in the context of lab-on-chip devices, where tightly packed droplets (or bubbles), typically one hundred micrometers in diameter, are manipulated within channels of a few hundred microns width.^25,31^ In such a confined environment, the applied stress, controlled by the flow rate, may be tuned to achieve a desired response of the droplet structures. Nonetheless, improper handling of these materials (such as by excessive stresses) may lead to droplet coalescence or fission,^32^ thus irretrievably altering the “grain” size and/or polydispersity of the double emulsion while also dramatically affecting the topological arrangement of the internal drops. Hence, understanding the fluid–structure interactions governing the physics at these scales is of paramount importance for precise manufacturing and correct functioning of these soft granular materials.

From a theoretical standpoint, numerical simulations represent the only viable strategy to study the multiscale physics of these systems, due to the complex structure of the equations involved.^14,33−41^ While efforts have been dedicated over the years to experimentally generate multicore emulsions with a high level of control,^9−11,16,42^ to date, no theoretical studies have investigated the transport of a HIPdE structure through a narrow channel by fully incorporating hydrodynamic interactions. This work aims precisely at filling this gap using an extended version of a lattice Boltzmann (LB) method recently proposed to study dense emulsions produced in microfluidic channels.^43,44^

Here, we numerically investigate, by means of two-dimensional simulations, the translocation of dense double-emulsion drops through a constriction whose design is inspired by a typical microfluidic setting. The numerical experiment sets off with a HIPdE encapsulating multiple tightly packed cores initially relaxed in a wide inlet channel, subsequently driven by a uniform fluid flow through a narrow channel, and finally relaxed in a wide outlet chamber. The emulsion is made of three fluid components—a dispersed droplet phase (A), an inter-droplet lubricating phase (B), and an external bulk fluid (C) (see Figure 1).

Our results show that shape and mechanical properties of the resulting emulsion are critically influenced by (i) the area fraction ϕ of the internal cores and (ii) the height *h*_s_ of the constriction with respect to the size of the HIPdE. During translocation, the double-emulsion drop undergoes dramatic shape deformations, which entail a considerable elongation of the shell combined with a substantial rearrangement of the cores. At low values of ϕ (generally smaller than ∼0.55), the emulsion collected in the outlet chamber essentially recovers the shape attained in the inlet channel, while at higher values of ϕ, the final shape considerably deviates from the initial one (before translocation). This behavior is evaluated in terms of quantitative parameters, such as circularity of the emulsion and energy dissipated during the crossing. Also, decreasing the height of the constriction favors deeper structural changes, whose dynamics is controlled by the ratio between viscous dissipation and the energy necessary to restore shape deformations. If the former dominates, substantial shape changes are inevitable. We finally note that our findings provide a precise mapping of observables, such as velocity gradients and topological changes within a dense emulsion, difficult to measure experimentally^45^ but essential to predict the dynamic behavior of a HIPdE. Notwithstanding, the question whether some aspects of the dynamical behavior of various types of granular aggregates, such as active^45−47^ and passive^10,48^ ones, may be universal remains open, and here, we make a step toward better general understanding of the behavior of a purely passive system.

The paper is organized as follows. In the next section, we shortly outline the numerical method used in this work, while further details can be found in refs (33, 33, 43). In Results section, we initially discuss the typical shape deformations of a dense emulsion observed within a constriction, and then, we focus on memory-like effects induced by the area fraction of the cores and height of the channel on the final shape of the emulsion. Some final remarks conclude the manuscript.

## Methods

The numerical model employed in this work is based on a recently proposed LB color gradient approach for multicomponent flows with near-contact interactions.^33,43,44^ Here, it is extended to simulate double emulsions with multicore morphology made of a three-component fluid—a droplet phase (A), an inter-droplet lubricating phase confined within a shell (B), and an external continuous phase (C) (see Figure 1).

This LB method is built starting from three sets of probability distribution functions (PDFs), whose evolution is governed by a discrete transport Boltzmann equation^49−53^
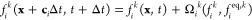
1

In the abovementioned equation, *f*_*i*_^*k*^ is the discrete distribution function (namely, the probability of finding a particle of the *k*th (*k* = A, B, C) fluid component at position **x** and time *t*) moving along the *i*th direction at a (discrete) velocity **c**_*i*_. The set of distributions streams along the directions identified by the lattice geometry, which is constructed with sufficient symmetry/isotropy to reproduce hydrodynamic moments up to the desired order. A nine-speed lattice in two dimensions (D2Q9) and 19 or 27 speeds in three-dimensions (D3Q19 and D3Q27), for example, hold moment isotropy up to the second order, enough to capture the physics of fluids up to the Navier–Stokes level.^49,54^ In this work, we performed two-dimensional simulations; thus, we opted for a D2Q9 LB model.

The collision operator Ω_*i*_^*k*^(*f*_*i*_^*k*^,*f*_*i*_eq^,*k*^) depends on the discrete set of PDFs and on the set of equilibrium distribution functions obtained by performing a Taylor expansion of the local Maxwell–Boltzmann distribution, up to the second order in the Mach number.^49^ Within the color gradient framework, it is typical to split the collision operator into three parts
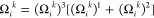
2where (Ω_*i*_^*k*^)^1^ stands for the standard relaxation operator of the set of distribution functions, (Ω_*i*_^*k*^)^2^ codes for the perturbation step, contributing to the buildup of an interfacial tension between immiscible components, and (Ω_*i*_^*k*^)^3^ is an anti-diffusion term, needed to guarantee the immiscibility between different fluid components and to minimize the thickness of the diffuse interface (more details can be found in refs (33, 43)). Finally, to arrest the coarsening and prevent the coalescence between neighboring droplets in close contact, a mesoscale repulsive force, essentially capturing the effect due to a surfactant adsorbed onto the interfaces, is included in the model. In previous studies,^33,43^ it has been shown that in the continuum limit, such an approach leads to a Navier–Stokes equation for a multicomponent system augmented with a surface-localized body force, which can be interpreted as a coarse-grained version of the short-range molecular forces acting at the nanometer and sub-nanometer scales.

This numerical approach provides a robust platform for the simulation of dense emulsions with double-emulsion morphology like those reported in Figure 1, which shows a qualitative comparison between two dense foam-like structures obtained experimentally (Figure 1a) and numerically (Figure 1b).

### Numerical Details

**Figure 2:**
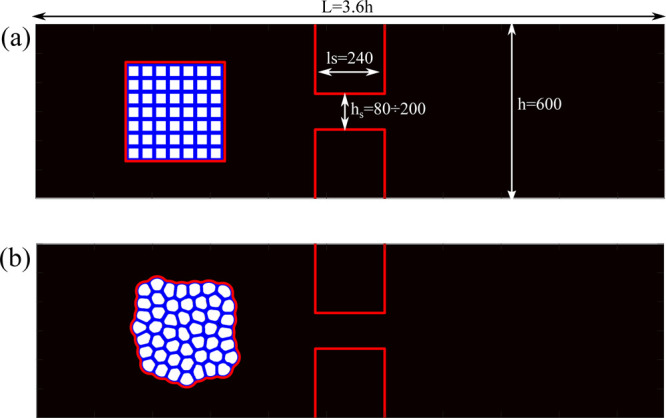
(a) Double emulsion with ϕ ≃ 0.9 and *N*_d_ = 49 is initialized as a checkerboard-like pattern within the inlet chamber. (b) Example of the structure of the emulsion attained after *t* = 5000 time steps. The height of the inlet and outer chambers is *h* = 600 lattice sites, while the total length of the microfluidic channel is *L* = 3.6*h*. The rectangular constriction is made of two opposite fluid-free squared regions (indicated by red lines) of length *l*_s_ = 240 lattice units, placed at a distance *h*_s_ (the height of the constriction) ranging from 80 to 200 lattice sites.

The simulation setup consists of a two-dimensional microfluidic channel made of an inlet reservoir followed by a thinner channel connected to a further downstream reservoir (see Figure 2).The height of the chambers is *h* = 600 lattice units and that of the constriction varies from *h*_s_ = 80 to *h*_s_ = 200 lattice units, while its length is *l*_s_ = 240 lattice units. At the walls, we impose a bounce-back rule for the distribution functions, while at the outlet, we employ absorbing (zero-gradient) boundary conditions.^51^ As mentioned above, the triple emulsion consists of fluid droplets (component A, white squares in Figure 2a) immersed within an inter-droplet continuous phase (component B, blue lines) surrounded by an external bulk phase (component C, black region outside the emulsion). The emulsion is initialized as a checkerboard-like pattern within the inlet chamber and relaxed for ∼5000 time steps, enough to achieve a configuration essentially stable over long periods of time. The number of the cores *N*_d_ has been varied from 25 to 49, while their diameter *d* has been varied from 36 to 40 lattice sites to simulate different packing fractions. With these numbers, the area fraction ϕ = *A*_d_/*A*_s_ ranges from 0.5 up to 0.9, where *A*_d_ is the area occupied by the cores and *A*_s_ is the area of the shell.

Note that the shape of the emulsion decisively depends on ϕ. Indeed, while at low ϕ, the drops and the external interface essentially keep their circular shape; at higher area fractions, inner cores are heavily squeezed and the external interface displays a multifaceted structure, tightly fitted to that of the nearby cores. This occurs since the fluid confined within the emulsion is almost entirely substituted by the cores. As also detailed in the following, the viscous dissipation is expected to hinder the relaxation of the shell, thus opposing to surface tension forces, which would restore the circular shape of the interfaces.

Notwithstanding, a crude estimation of the typical size of the shell (such as its diameter) can be computed by simply summing the diameters of the cores along one direction, say, the vertical one, where one has about seven cores. Hence, the ratio *h*_s_/*D*, where D ≃∑_*i*_*d*_*i*_ is the diameter of the emulsion, ranges approximately between 0.3 and 0.8.

The physical parameters have been chosen as follows. The kinematic viscosity of the dispersed phase and of the shell is ν_B_ = ν_C_ = 0.167, while that of the droplet phase has been set to ν_A_ = 0.05. The surface tensions between different components have been set to σ_AB_ = 0.02, σ_BC_ = 0.05, and σ_AC_ = 0.01. Such values are reported in simulation (lattice) units (details about the conversion in physical units^51^) and are chosen to match viscosity ratios and surface tension ratios of water (A), hexadecane (B), and fluorocarbon fluid (C), mixtures often employed in microfluidic experiments (see Figure 1).^10^

## Results

We initially discuss the typical behavior of a dense emulsion flowing within a narrow channel, focusing on shape deformations of the drops and structure of the velocity field. Afterward, we study the dynamic response of the emulsion, evaluated by varying the number *N*_d_ of internal drops and height *h*_s_ of the constriction.

### Shape Deformations and Velocity Field within a Narrow Channel

**Figure 3:**
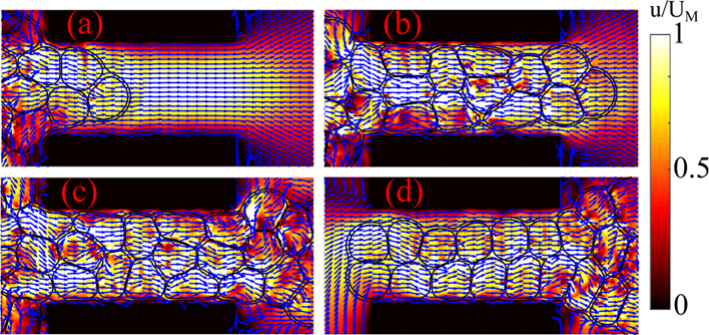
Translocation of a dense emulsion within a narrow channel. Here, ϕ ≃ 0.9, *N*_d_ = 49, and *h*_s_ = 120, and the black regions represent the walls. A uniform velocity field pushes the emulsion rightward, causing a flow of drops approximately arranged in a two-row structure (a). Afterward, the drops gradually fill the constriction, giving rise to a highly compact foam-like body, which, once out of the channel, expands and relaxes (b–d). The velocity field is generally rather uniform within the constriction, whereas it is irregular in the drops out of the channel. The color bar represents the ratio of the magnitude of the velocity field *u* with respect to its maximum value *U*_M_.

In Figure 3a–d, we show an example of translocation of a dense emulsion. In our numerical experiment, a uniform velocity profile, of speed *U*_in_ = 2 × 10^–3^ (in simulation units), is imposed at the inlet, which pushes the emulsion, of ϕ ≃ 0.9 and *N*_d_ = 49, within a narrow constriction of height *h*_s_ = 120 lattice units. A parabolic profile of the velocity starts at *x* ∼ 0.15*L* within the inlet chamber and fully develops within the constriction, where the average speed is approximately given by (*A*_in_/*A*_con_)*U*_in_, with *A*_in_ and *A*_con_ being the cross-sections of the inlet and constriction, respectively. The value of the velocity ensures that capillary and Reynolds numbers remain well within the typical range of microfluidic experiments ( and , respectively).

Once the emulsion approaches the constriction, the leading edge of the outer shell gradually deforms, while the internal cores located nearby undergo a series of mutual rearrangements necessary to flow within the channel (Figure 3a). Note that due to the high area fraction, the shape of the cores is generally far from the circular one, and at this stage of the translocation, it is only mildly affected by the constriction. Afterward, the emulsion gradually fills the channel (Figure 3b,c), giving rise to flowing metastable clusters of drops approximately arranged in a two-row structure. Once the leading edge of this structure has left the constriction, the cores at the forefront slowly unpack, while those in the channel assemble in a compact foam-like flowing material made of highly deformed drops separated by thin fluid films. During such a process, the velocity field exhibits two distinct patterns: an irregular and chaotic structure within the cores located out of the channel and a more uniform and ordered one within. The former is essentially due to the coupling between the velocity field and interfaces of the cores, an effect significantly mitigated in the constriction where a heavier flow, caused by the confined geometry, drags the drops collectively rightward at a higher speed.

The translocation clearly entails a remarkably complex rearrangement of the material, whose shape is profoundly influenced by the geometry of the channel. However, to what extent the confined environment can reshape the emulsion? In other words, can the structural changes observed in this dense emulsion become permanent, thus resulting in a soft material with mechanical and topological properties radically different from the ones exhibited initially? The next section is precisely dedicated to explore these issues.

### Permanent Shape Deformations in Highly Dense Emulsions

To assess the effect produced by the inner drops on the shape of the emulsion once the translocation has occurred, we have performed a series of simulations by varying the number *N*_d_ of internal cores and their area fraction ϕ. As mentioned above, the numerical experiment essentially consists of an almost equilibrated dense emulsion placed at the inlet and pushed within a constriction by a uniform fluid flow. The material is finally collected in the outlet chamber and is let to relax for a sufficiently long period of time.

**Figure 4:**
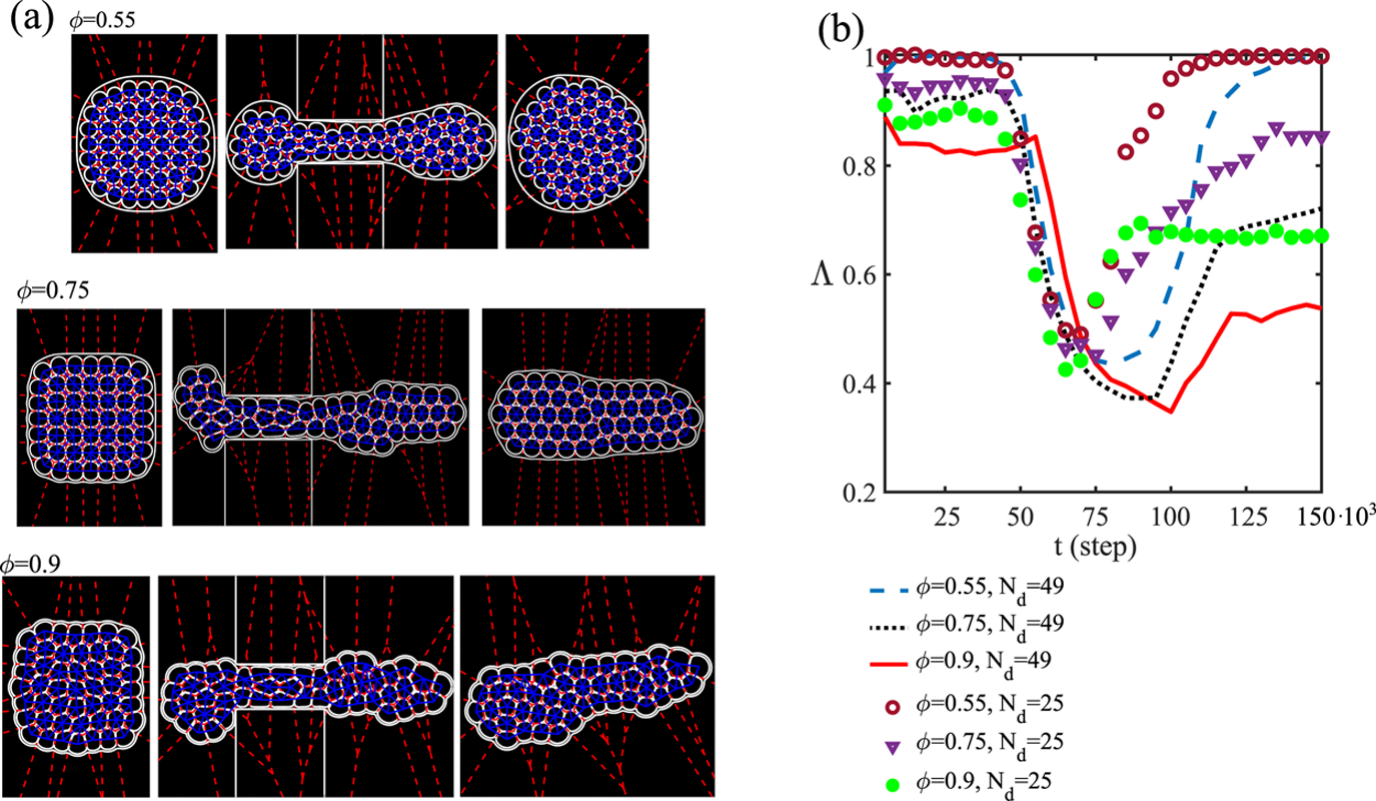
(a) Translocation dynamics of a dense emulsion for ϕ = 0.55 (top), ϕ = 0.75 (middle), and ϕ = 0.9 (bottom). In all cases, *N*_d_ = 49 and *h*_s_/D ≃ 0.45. Once the emulsion crosses the constriction, the resulting shape observed in the outlet chamber crucially depends on the area fraction ϕ. At low ϕ, the emulsion expands and springs back close to its original shape; at increasing ϕ, deformations become permanent. Snapshots are taken at *t* = 25, 90, and 150 × 10^3^ steps. Blue lines connecting the centers of neighboring droplets represent the Delaunay triangulation, while red dotted lines represent the Voronoi decomposition. (b) Time evolution of the circularity Λ for different values of ϕ and *N*_d_. At low values of ϕ, the circular shape of the emulsion is basically restored after translocation, while at increasing ϕ, Λ considerably decreases, up to half of its initial value.

In Figure 4a, we show the results of three simulations obtained for ϕ = 0.55 (top, movie M1.avi), ϕ = 0.75 (middle, movie M2.avi), and ϕ = 0.9 (bottom, movie M3.avi), with *N*_d_ = 49 and diameter of the cores equal to *d* = 36 (top), *d* = 38 (middle), and *d* = 40 (bottom). Since *h*_s_ = 120 lattice units, one has *h*_s_/D approximately equal to 0.45. Once the flow is switched on, the emulsion crosses the constriction undergoing considerable shape deformations, basically akin to those described in the previous section. However, the shape of the resulting material collected in the outer chamber crucially depends on the area fraction ϕ occupied by the cores and on the ratio *h*_s_/*D*.

Keeping *h*_s_/*D* fixed at ≃ 0.45, one can basically distinguish two relevant scenarios. Indeed, if ϕ = 0.55, the final shape resembles very closely the initial one observed before the translocation, whereas at increasing values of ϕ, the double-emulsion drop generally exhibits a highly elongated structure considerably different from the starting configuration. Such shape deformations are found to persist over long periods of time and can be considered permanent rather than temporary.

The departure of the emulsion from its circular shape can be monitored by computing the circularity of the external shell, defined as Λ = 4π*A*/*P*^2^, where *A* is the area of the shell and *P* is its perimeter. This quantity ranges between 1 (circular shape) and 0 (needle-like geometry).In Figure 4b, the time evolution of Λ for different values of ϕ and *N*_d_ and for *h*_s_ = 120 is shown. At low values of ϕ (such as ϕ ∼ 0.55 and *N*_d_ = 49, hollow circles, or *N*_d_ = 25, dashed line) Λ goes back to ∼1 at late times, indications that deformations are negligible. For increasing values of ϕ (∼0.75, dotted line and hollow triangles, and ∼0.9 continuum line and filled circles), after the translocation, Λ attains values well below 1 kept essentially constant for a long period of time, thus quantitatively proving that shape changes can be considered essentially permanent. Note that finally, regardless of the area fraction, the minimum of Λ sets at roughly 0.4, basically because the height *h*_s_ of the constriction has been kept constant in this set of simulations.

**Figure 5:**
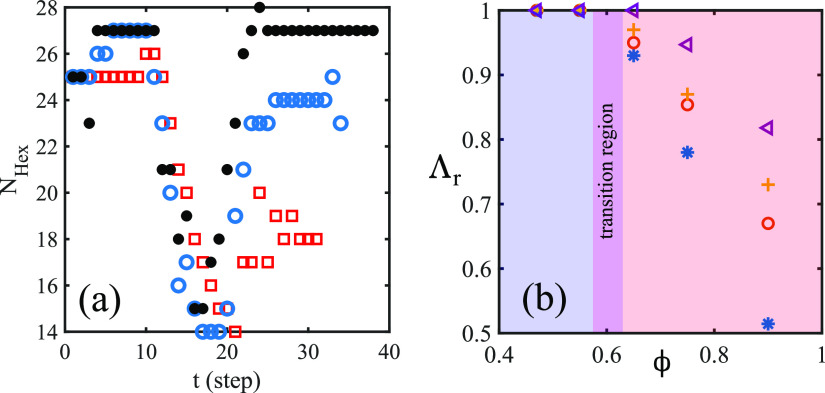
(a) In this figure, we show the time evolution of the number of hexagonal clusters *N*_Hex_, computed by a Delaunay triangulation, observed during the translocation for ϕ ≃ 0.55 (black circles), ϕ ≃ 0.75 (empty circles), and ϕ ≃ 0.9 (squares). Like Λ, *N*_Hex_ exhibits a minimum, approximately equal for all values of ϕ, within the constriction, and attains a late time value, which decreases when ϕ augments. (b) Here, we show the behavior of the residual deformation Λ_r_ for different values of ϕ, *N*_d_, and *h*_s_. In particular, *h*_s_ = 200 and *N*_d_ = 25 (triangles), *h*_s_ = 200 and *N*_d_ = 49 (plusses), *h*_s_ = 120 and *N*_d_ = 25 (circles), and *h*_s_ = 120 and *N*_d_ = 49 (asterisks). Λ_r_ is computed as the time average of the values of Λ obtained once the translocation is completed. As long as ϕ < 0.6, shape deformations are negligible, while for larger values of ϕ, Λ_r_ decreases faster for smaller values of *h*_s_ (i.e., narrower channels).

The complex topological rearrangements of the droplets can be quantitatively assessed by monitoring the time evolution of the number of hexagonal clusters *N*_Hex_ assembled in the process. This quantity, computed using a Delaunay triangulation, provides insights into how far such clusters are from the ordered hexagonal structure typical of a solid material. Like the circularity, *N*_Hex_ exhibits a minimum, roughly equal for all values of ϕ, when a large portion of the emulsion is within the constriction, whereas it attains a late time value, which decreases for increasing values of ϕ, once the translocation is completed (see Figure 5a). This result suggests that under extreme confinement, the crystal-like ordering dramatically drops, while it is only partially restored once the emulsion has relaxed in the outer chamber. In other words, the emulsion has undergone a permanent modification in its shape after the crossing within the narrow channel. We incidentally note that unlike this system, fully hexagonal clusters of droplets can survive within channels. This is the case, for example, of microfluidic crystals,^25^ where droplets (or bubbles) are fed into the channel at a constant rate from a single tube without fluctuations, thus quickly jumping into local energy minima with a crystal-like configuration.

Finally, the effect produced by the constriction can be evaluated by computing the residual deformation Λ_r_, calculated as a time average of the values of Λ obtained once the translocation ends (see Figure 5b). Such deformation is found to remain essentially constant and close to unity for ϕ ≤ 0.6 regardless of the values of *h*_d_, whereas it decreases down to ∼0.5 for higher values of ϕ and for narrower sizes of the channel. In this regime, the decrease is steeper for diminishing values of *h*_d_, a result proving, once again, that heavy shape changes observed during the translocation become stable and permanent for long periods of time.

### Dynamical Characterization

The results discussed above show that the transition from a Newtonian behavior, in which shape changes are negligible, toward a viscoelastic one, in which on the contrary they become permanent, is observed for high values of ϕ (larger than 0.6), a regime in which fluid interfaces occupy a large portion of the emulsion. It is thus crucial to understand the role played by such interfaces and to what extent they affect the mechanical properties of the emulsion.

**Figure 6:**
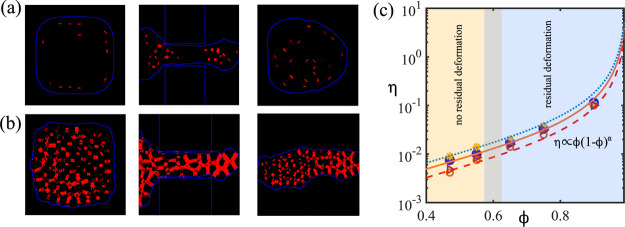
(a,b) Map of the magnitude of the velocity gradients within the emulsion for ϕ ≃ 0.55 (a) and ϕ ≃ 0.9 (b). The spots highlighted in red represent regions in which |∇**u**| > |∇**u**_th_|. (c) Dissipation parameter η (i.e., viscous dissipation vs surface tension work) is plotted as a function of ϕ and for different values of the ratio *h*_s_/*D*, in particular *h*_s_/D ∼ 0.6 (open circles), *h*_s_/*D* ∼ 0.8 (open triangles), *h*_s_/*D* ∼ 0.35 (filled circles), and *h*_s_/*D* ∼ 0.5 (asterisks). Dotted, continuous, and dashed lines represent the best fit to asterisks, filled circles, and open circles, respectively.

In Figure 6a,b, we show a map of the magnitude of the velocity gradients within the emulsion for ϕ ≃ 0.55 (a) and ϕ ≃ 0.9 (b). The spots highlighted in red represent regions in which |∇**u**| > |∇**u**_th_|, where  and |∇**u**_th_| ∼ 10^–3^ is a threshold below which shear effects are negligible. The choice of the threshold is motivated as follows. By defining a grid capillary number as Ca_l_ = μ|∇*u*|Δ*x*/σ, with μ ∼ 0.1, Δ*x* = 1, σ = 0.01, and |∇*u*| = 10^–3^, we obtain Ca_l_ ∼ 10^–2^. Hence, if |∇*u*| < 10^–3^, it is reasonable to assume that the local shear stresses are negligible with respect to the surface tension forces. High values of |∇**u**| are generally concentrated between co-moving interfaces in close contact, and only at high ϕ, they form an interconnected path running through the tangle of the thin films sustained by the plateau of the droplet’s interfaces. Remarkably, once the emulsion has left the constriction, high gradients survive only if ϕ ≃ 0.9, whereas they are basically negligible for lower values.

Since the interfaces are the source of the viscous dissipation in a fluid mixture, one may expect that in the high-ϕ regime, viscous forces become the dominant contribution with respect to the one stemming from surface tension, which opposes shape deformations. This is evaluated by introducing a dissipation parameter, η = ⟨*P*_**Ψ**_⟩/⟨*P*_**σ**_⟩, which essentially compares the loss of energy due to viscous dissipation to the work performed by surface forces to bring the droplets back to their spherical shape once the translocation is complete, thus favoring the relaxation of the external shell. Here, ⟨*P*_**Ψ**_⟩ and ⟨*P*_**σ**_⟩ represent time-averaged values of viscous and surface energy rates, respectively, defined as
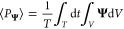
3and
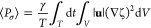
4In eq 3, the local (time–space) viscous function **Ψ** is defined as
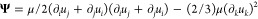
5where μ is the dynamic viscosity,  is the local phase field varying between 1 (bulk of fluid A) and −1 (bulk of fluid B), and the term γ(∇ζ)^2^, with γ ∼ σ_AB_, represents an interfacial free energy contribution, overall akin to the one of Landau theory of multicomponent fluids usually given by (*k*/2)(∇ζ)^2^, with *k* being the elastic constant.^51^ Finally, in both equations, *V* is the volume occupied by the emulsion and T is the time integration interval.

In Figure 6c, η is plotted as a function of ϕ and for different values of the ratio *h*_s_/*D*. Its behavior is essentially independent of *h*_s_/*D* but steadily increasing with ϕ. The growth is linear for ϕ < 0.6, where the emulsion behaves as a Newtonian fluid, and follows a non-linear trend for higher values of ϕ, the region where viscoelastic features emerge. In this regime, η ≥ 10^–2^, a value above which the viscous dissipation, converted into thermal heating due to the high velocity gradients localized at the fluid interfaces, becomes a non-negligible fraction of the total surface energy. It is best fitted with a power law of the form *A*ϕ(1 – ϕ)^α^, where *A* is a constant and −3/2 ≤ α ≤ −4/3. This trend is reminiscent of the viscosity–concentration relation observed in packed emulsions (theoretically predicted by Bullard et al.^55^), which, unlike analogous soft materials displaying a first-order solid-to-liquid transition above a critical threshold,^30^ follows a continuous power law behavior.

These results show that the ratio between the internal viscous forces (extensional force) and the surface tension (retraction/restoring force) controls the dynamics of a dense emulsion during the compression/expansion process. Indeed, while internal viscous forces retard the expansion of the external shell, the surface tension opposes the effect of the extensional stresses. This competition, together with the degree of confinement set by the constriction, modulates the final shape of the core emulsion.

## Conclusions

In this work, we have numerically investigated, using LB simulations, the dynamic behavior of double-emulsion drops with close-packed inner droplets crossing a narrow channel. Our system is a ternary fluid mixture, in which a dispersed droplet phase is immersed in an inter-droplet lubricating one, in turn surrounded by an external bulk fluid. The double-emulsion drops are initialized in an inlet chamber, subsequently driven by a uniform flow within a constriction, and finally collected in an outlet chamber, where they are let to relax.

Our results show that the shape of the double-emulsion drop obtained downstream crucially depends on (i) the area fraction of the internal droplets and (ii) the height of the narrow channel with respect to the typical size of the double-emulsion drop. For fixed values of height, but significantly smaller than the diameter the drop, we basically distinguish two scenarios. If the area fraction occupied by the cores remains below ∼0.55, once the translocation within the channel is completed, the double-emulsion drop essentially recovers the quasi-spherical shape attained before the crossing. On the contrary, for higher values of the area fraction, shape deformations induced by the constriction become permanent, and the drop generally attains a heavy elongated shape, in which highly packed inner droplets are separated by thin films of fluid. The deviation from the spherical shape is measured in terms of the circularity Λ, which is found to fall up to 0.5 for very dense emulsions (ϕ ≃ 0.9) and very narrow constrictions (*h*_s_/D ≃ 0.5 or lower values). The transition from a Newtonian fluid behavior toward a non-Newtonian one is captured with high accuracy using the dissipation parameter η, a dimensionless number representing the loss of energy due to viscous dissipation with respect to the work performed by surface forces necessary to round up deformations. This quantity follows a power law behavior, approximately linear for ϕ < 0.55 and highly non-linear for higher values; the latter is an indication that at a large area fraction, viscous forces dominate over surface tension ones, an effect very likely due to fluid interfaces occupying a large portion of the emulsion.

In summary, we find that our system develops memory-like effects induced by the combined action of confinement and viscous dissipation. Understanding the behavior of soft granular clusters, such as the double-emulsion drops studied here, can be of interest for the generation of compartmentalized capsules and vesicles useful, for example, in drug delivery. Their structure can give rise to a significant amount of internal dissipation, thereby considerably affecting the way they flow in complex geometries, such as obstacles and orifices. Under strong confinement, events such as topological transitions, coalescence, and rearrangement of droplets may crucially affect the stability and lead to morphological transitions,^25,31^ such as fragmentation of the outer shell (breakup of a single HIPdE into multiple HIPdEs) or coalescence of the inner droplets. Control of such systems is also crucial for the preparation of dense emulsions and double emulsions as templates of porous materials with desired porosity and/or pore arrangement (e.g., ordered vs disordered).^1^

Besides their interest on its own, such largely unexplored complex states of flowing matter could also raise interest in fields such as biology and medicine. In the former case, our numerical experiments could be seen as a droplet microfluidic analogue of compression–relaxation experiments involving aggregates of cells (the inner cores in our emulsion).^46^ In the latter context, modeling the migration of cell clusters within confined environments, such as across capillaries, is essential to assess how morphological changes affect their motion, a phenomenon of particular relevance in pathological diseases such as invasion and metastasis of cancer cells.^56,57^

## Supporting Information Available

Translocation dynamics of a HIPdE with ϕ ≃ 0.55 (AVI)
Translocation dynamics of a HIPdE with ϕ ≃ 0.75 (AVI)
Translocation dynamics of a HIPdE with ϕ ≃ 0.9 (AVI)

## Supplementary Material

la1c01026_si_001.avi

la1c01026_si_002.avi

la1c01026_si_003.avi
